# Editorial Staff of the Independent Practitioner

**Published:** 1881-10

**Authors:** 


THE EDITORIAL STAFF OF THE INDEPENDENT PRACTITIONER will not be selected in haste, but with great care, and may not be announced before January, 1882. Our object is to secure the *best talent*, and make our publication *the journal*. We will, therefore, strenuously avoid men who have "axes to grind," or enemies to denounce, but seek gentlemen who are lovers of *science* and *truth*, and can say much in few words. There is more written for the sake of euphony than to convey new thought; such articles the busy Practitioner has not the time to read.

